# A Subunit Vaccine Based on E2 Protein of Atypical Porcine Pestivirus Induces Th2-type Immune Response in Mice

**DOI:** 10.3390/v10120673

**Published:** 2018-11-28

**Authors:** Huawei Zhang, Wei Wen, Genxi Hao, Huanchun Chen, Ping Qian, Xiangmin Li

**Affiliations:** 1 State Key Laboratory of Agricultural Microbiology, Huazhong Agricultural University, Wuhan 430070, Hubei, China; azhangzhizhong@126.com (H.Z); 15827354689@163.com (W.W.); chzhx521@163.com(G.H.); chenhch@mail.hzau.edu.cn (H.C.); 2 Laboratory of Animal Virology, College of Veterinary Medicine, Huazhong Agricultural University, Wuhan 430070, Hubei, China; 3 Key Laboratory of Development of Veterinary Diagnostic Products, Ministry of Agriculture, Wuhan 430070, China; 4 Key Laboratory of Preventive Veterinary Medicine in Hubei Province, The Cooperative Innovation Center for Sustainable Pig Production, Wuhan 430070, Hubei, China

**Keywords:** atypical porcine pestivirus, E2 protein, subunit vaccine, Th2-type immune response

## Abstract

An atypical porcine pestivirus (APPV) causing congenital tremor type A-II in piglets was identified in China in 2016. An increased number of cases of APPV have been reported in various countries all over the world since 2015. This study aimed to develop an effective subunit vaccine against APPV based on the E2 protein, which is the main immunogenicity protein of APPV. In this study, E2 protein was successfully expressed by the baculovirus expression system. E2 protein was confirmed by Western blot assay, which showed that E2 protein possesses N-linked glycosylation sites. The immunogenicity of E2 subunit vaccine was evaluated in mice. The E2 protein emulsified with ISA 201VG adjuvant induced significantly higher levels of APPV-specific antibodies and elicited stronger lymphocyte proliferative responses and higher interleukin-10 secretion than those of the E2 protein emulsified with IMS 1313VG adjuvant. This observation indicates that the E2 subunit vaccine induces a Th2-type immune response. Our results showed that E2 protein can be developed as a safe and effective subunit vaccine for the control of APPV infection.

## 1. Introduction

Porcine congenital tremor (CT) is a disease of newborn piglets. It is characterized by serious pathological damage to the brain and spinal cord and manifests as tremors of the head and limbs [1,2]. Kinsley et al. reported CT for the first time in 1922 [1]. Since then, CT has widely emerged in the pig industry worldwide. Research has divided CT into five subgroups on the basis of cause, and type A-II cases have been considered with the causative agent [1]. Arruda, B.L. et al. have reported that CT type A-II is associated with atypical porcine pestivirus (APPV) [2], which is a novel porcine pestivirus first found by next-generation sequencing in 2015 [3].

APPV features a single-stranded positive-sense RNA genome of approximately 11 kb and a member of the Pestivirus genus [4]. APPV consists of a single open reading frame flanked by 5′- and 3′-untranslated regions encoding a 3635-amino-acid polyprotein containing four structural proteins (Npro, C, Erns, E1, and E2) and eight nonstructural proteins (P7, NS2, NS3, NS4A, NS4B, NS5A, and NS5B) [3]. Pestiviruses contain three envelope glycoproteins (Erns, E1, and E2). The E2 protein is the principal component of APPV protein; it spans 241 amino acids in length and is significantly shorter than the E2 protein of other pestiviruses [3]. The E2 glycoprotein of other pestiviruses (such as classical swine fever virus (CSFV)) can elicit a strong immune response and confer protection after CSFV infection [5].

CT type A-II outbreaks have been reported worldwide [4,6,7,8,9,10,11,12,13,14]. Newborn piglets infected with APPV die by starvation or suffer from retarded growth. Outbreaks of CT type A-II incurred economic losses in domestic pigs globally [1]. However, no effective vaccine nor drug can either prevent or cure this disease. Bioinformatics analysis showed that several B and T cell epitopes can be found in the APPV E2 protein, indicating that the E2 protein is immunogenic. Thus, the E2 protein of APPV is an ideal target for the development of genetically engineered vaccine against APPV.

In the present study, a recombinant baculovirus expressing APPV E2 protein was constructed to evaluate the immune responses of the E2 protein of APPV in mice. The E2 protein emulsified with ISA 201VG adjuvant induced significant humoral and cellular immune responses. Hence, the E2 subunit vaccine is a promising candidate vaccine against APPV.

## 2. Materials and Methods

### 2.1. Construction of Recombinant Baculoviruses

Plasmid pEASY-BLUNT-APPV E2 (GenBank No.KY652092.1) containing the honeybee melittin signal peptide at the N terminus and 6 × His tag at the C terminus without endogenous transmembrane domain was constructed by overlap polymerase chain reaction and maintained in our laboratory. DNA fragments, as templates, were amplified and subcloned into the pFBD vector [5], which included three independent gene expression cassettes. To obtain the recombinant construct pFBD-3-APPV-E2, we performed three separate PCR reactions using the plasmid pEASY-BLUNT-APPV E2 as the template. The three PCR products were inserted into the pFBD vector. Finally, the transfer vector pFBD-3-APPV-E2 encoding three copies of APPV-E2 was obtained; each APPV-E2 copy was obtained within its own expression cassette, including a polyhedrin promoter and a transcription termination sequence [5]. Finally, the transfer vector pFBD-3-APPV-E2 expressing three copies of APPV E2-6×His was constructed. The recombinant transfer vector was confirmed through sequencing. The recombinant baculovirus, Ac-3-APPV-E2, was generated by the viral plaque assay using the Bac-to-Bac system (Invitrogen, Carlsbad, CA, USA) in accordance with the manufacturer’s instructions.

### 2.2. Detection of APPV E2 Protein Expression and Purification of E2 Protein

The Sf9 cells (ATCC^®^ CRL-1711™) were seeded at a concentration of 1 × 10^6^ cells/well into 6-well tissue culture plates and were infected with the recombinant baculovirus (Ac-3-APPV-E2) at a multiplicity of infection (MOI) of 1. After cultivation at 27 °C for 60 h, the supernatant and cell lysate were collected and analyzed by Western blot. The samples were separated using 12% sodium dodecyl sulfate polyacrylamide gel electrophoresis (SDS-PAGE) and then transferred onto polyvinylidene difluoride membranes. The membrane was blocked with 5% skim milk in phosphate buffer saline with Tween (PBST) (137 mM NaCl, 2.7 mM KCl, 10 mM Na_2_HPO_4_, 2 mM KHPO_4_, and 0.05% Tween-20) and incubated with His monoclonal antibodies and horseradish peroxidase (HRP)-conjugated goat anti-mouse IgG (Boster Biological Technology, Wuhan, China). After washing with PBST, protein bands were detected using the enhanced chemiluminescence system and analyzed using the Image Lab software 4.0.1. At the same time, the samples were digested with peptide-N-glycosidase F (PNGase F) (NEB, Ipswich, MA, USA), which was used to detect whether the E2 protein was modified by N-glycosylation. Then, the treated samples were analyzed by Western blot.

To purify the APPV E2 protein, High Five cells were infected with Ac-3-APPV-E2 recombinant baculovirus at a MOI of 0.0001. At 120 h after infection, the culture supernatants were centrifuged at 12000 rpm for 30 min and filtered using 0.22 µm filters to remove residual cell debris. APPV E2 protein was purified by AKTA protein purification apparatus through a nickel affinity chromatography column (GE, Chicago, IL, USA) in accordance with the manufacturer’s instructions. Then, the purified E2 protein was confirmed by 12% SDS-PAGE and quantified using a BCA protein assay kit.

### 2.3. Immunization of Mice

Animal experiments were performed in accordance with the protocols approved by the animal ethical and welfare committee of Huazhong Agricultural University (ethical numbers: N0. 42000600025312). The purified APPV E2 protein was emulsified with ISA 201VG adjuvant (Seppic, Paris, France) at a ratio of 1:1 (*w*/*o*/*w*) or IMS 1313VG adjuvant (Seppic, Paris, France) at a ratio of 1:1 (*w*/*w*) in accordance with the manufacturer’s instructions. Female BALB/c SPF mice aged 6-weeks old were purchased from Huazhong Agricultural University Experimental Animal Center and randomly divided into three groups of eight mice. Group A, the negative control group, was immunized with 200 µL PBS. Group B was intramuscularly vaccinated with 200 µL of the solution containing 40 µg of APPV E2 protein emulsified with ISA 201VG adjuvant. Group C was intramuscularly vaccinated with 200 µL of the solution containing 40 µg of APPV E2 protein emulsified with IMS 1313VG adjuvant. All mice were injected with the same dose of vaccine as booster immunization at 14 days post-primary immunization (dpi). Serum samples were collected at 0, 14, 28, and 42 dpi and stored at −20 °C until use.

### 2.4. Antibody Response

Anti-E2 IgG and IgG isotyping in the serum samples was performed by indirect enzyme-linked immunosorbent assay (ELISA). Ninety-six-well flat-bottomed plates were coated with purified E2 protein (100 ng/well) diluted in 0.1 M carbonate/bicarbonate buffer (pH 9.6). The plate was washed thrice and blocked with PBST (1% bovine serum albumin, PBS, and 0.05% Tween-20) at 37 °C for 1 h. After washing with PBST thrice, the serum samples diluted with PBST (1:400) were added to the wells and incubated at 37 °C for 1 h. The plate was washed again and incubated with HRP-conjugated goat anti-mouse IgG (1:10,000), IgG1 (1:10,000), IgG2a (1:5000), and IgG3 (1:5000) (ABclonal, Woburn, MA USA). Finally, total IgG and IgG isotyping titers were determined at 630 nm.

### 2.5. Lymphocyte Proliferation Assay

To evaluate T lymphocyte proliferation, the splenic lymphocytes were isolated from immunized mice using the lymphocyte isolation reagent (TBD, China). Lymphocytes (4 × 10^6^ cells/mL) were seeded into a 96-well plate with 100 µL Roswell Park Memorial Institute (RPMI) 1640 containing 10% fetal bovine serum (FBS) and stimulated with concanavalin A (10 µg/mL), purified E2 protein (10 µg/mL), and 100 µL RPMI 1640 containing 10% FBS. After culturing at 37 °C for 72 h, 20 µL MTS (5 mg/mL in PBS) was added to every well, and culturing was resumed at 37 °C for 4 h. The stimulation index (SI) was calculated in accordance with the formula: SI = (OD values of immunized groups − OD values of blank control) / (OD values of negative control group − OD values of blank control).

### 2.6. Cytokine Assays

Splenic lymphocytes were isolated and resuspended in RPMI 1640 medium supplemented with 10% FBS. Cell densities were adjusted to 4 × 10^6^ cells/mL and plated in a 24-well flat-bottom tissue culture plate with 10 µg/mL purified E2 protein. After 48 h of incubation at 37 °C, the cell-free supernatant was removed and detected using commercially available mouse interferon (IFN)-γ, interleukin (IL)-2, interleukin (IL)-4, and interleukin (IL)-10 ELISA kit in accordance with the manufacturer’s protocol. The concentrations of different cytokines were determined by the standard curve.

### 2.7. Statistical Analysis

Statistical analysis was performed by one-way ANOVA through GraphPad Prism software (GraphPad Software Inc., La Jolla, CA, USA). *p* < 0.05 was considered statistically significant.

## 3. Results

### 3.1. Expression of Recombinant Protein

**Figure 1:**
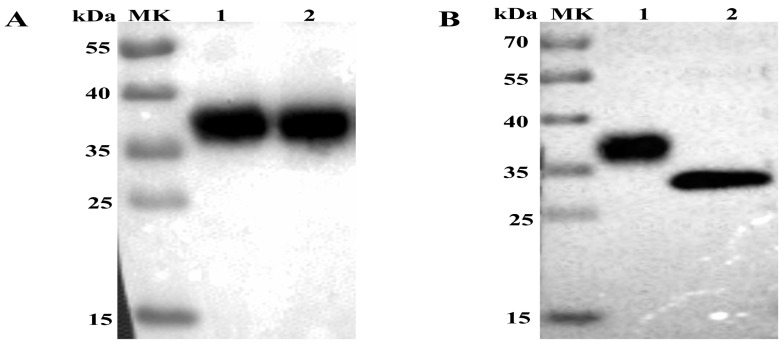
Western blot analysis of the expression of atypical porcine pestivirus (APPV) E2 protein. (**A**) Western blot analysis of APPV E2 protein expressed in Sf9 cells infected with the recombinant baculovirus AC-3-APPV-E2. Lane 1: Culture supernatant of sf9 cells infected with Ac-3-APPV-E2; Lane 2: Lysates of Sf9 cells infected with Ac-3-APPV-E2. (**B**) APPV E2 protein digested with PNGase F by Western blot analysis. Lane 1: APPV E2 protein as normal control; Lane 2: APPV E2 protein digested with PNGase F.

The supernatant and cell lysate were derived from sf9 cells infected with Ac-3-APPV-E2 and analyzed by Western blot. A single protein band with a molecular mass of approximately 36 kDa (Figure 1A) can be observed in the supernatant and lysates by anti-His monoclonal antibodies. However, no specific protein band was detected in sf9 cells (normal control). Results indicated that the APPV E2 protein was successfully expressed not only in the sf9 cell but also secreted into the supernatant fluid. The predicted molecular weight of the APPV E2 protein was 28.5 kDa, which is lower than that of the experimental result. We treated the E2 protein with PNGase F to verify whether the former is a glycoprotein. As a result, the treated E2 protein exhibited a decreased molecular weight relative to that of the normal control (Figure 1B), confirming that the APPV E2 protein is a glycoprotein.

### 3.2. Purification of APPV E2 Glycoprotein

**Figure 2:**
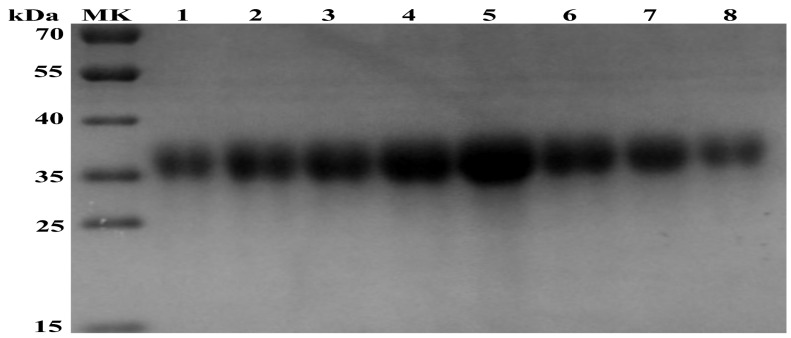
SDS-PAGE analysis of the purified recombinant APPV E2 protein. Lanes 1–8, purified recombinant APPV E2 protein eluted with five column volumes of 80 mM imidazole.

To obtain a purified APPV E2 glycoprotein, we infected High Five cells with Ac-3-APPV-E2. At 120 h after infection, the extant E2 glycoprotein in the supernatant was harvested by centrifugation. Then, the APPV E2 protein was purified by Ni-NTA chromatography. On the basis of the UV absorption peak value of the AKTA protein purification apparatus, recombinant E2 protein was eluted with five column volumes of 80 mM imidazole (Figure 2).

### 3.3. Specific Antibodies Elicited by E2 Protein Immunization

**Figure 3:**
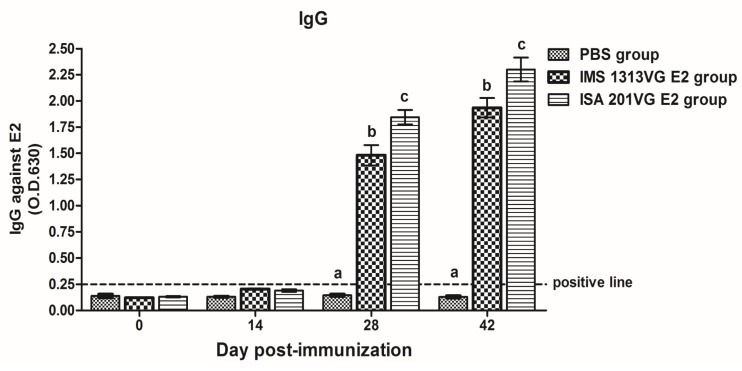
APPV E2-specific IgG detected by indirect ELISA at different dpi. Data represent the mean ± standard error of mean (SEM). Different letters (a, b, c) indicate a statistically significant difference between different experimental groups (*p* < 0.05).

As shown in Figure 3, the serum IgG titers of E2-IMS 1313VG and E2-ISA 201VG groups were significantly higher than those of the negative control group. Following booster immunization, E2-IMS 1313VG and E2-ISA 201VG groups exhibited increased antibody levels at 28 dpi. The levels of APPV E2-specific IgG antibody for Groups B and C displayed peak titers at 42 dpi with mean OD values (2.301 and 1.935, respectively).

**Figure 4:**
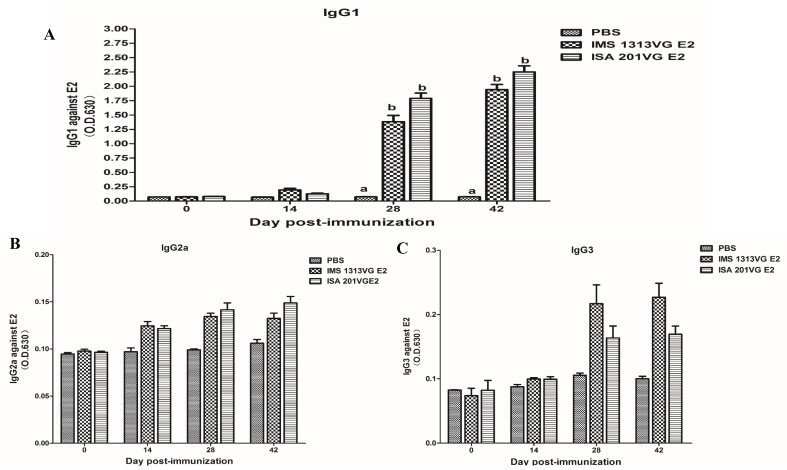
IgG subtype in serum samples tested using indirect ELISA. (**A**) APPV E2-specific IgG1 level. (**B**) APPV E2-specific IgG2a level. (**C**) APPV E2-specific IgG3 level. Data represent the mean ± SEM. Different letters (a, b) indicate a statistically significant difference between different experimental groups (*p* < 0.05).

Subtype IgG levels (IgG1, IgG2a, and IgG3) were measured by indirect ELISA to analyze the immune response profiles at 0, 14, 28, and 42 dpi. As shown in Figure 4, the mice immunized with APPV E2 protein induced the highest expression level of IgG1. The APPV E2-specific IgG2a and IgG3 levels were not detected at 0, 14, 28, and 42 dpi. The E2-IMS 1313VG and E2-ISA 201VG groups displayed peak IgG titers at 42 dpi. Therefore, these results indicate that Th2 type-dominant immune response was induced in mice.

### 3.4. Lymphocyte Proliferative Response

**Figure 5:**
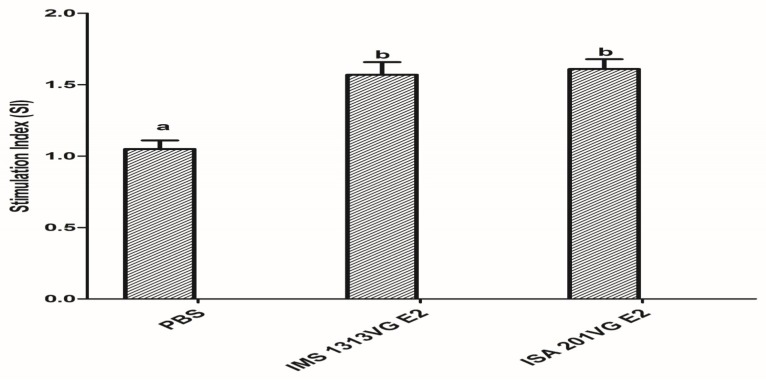
Detection of lymphocyte proliferative responses. Data represent the mean ± SEM. Different letters (a, b) indicate a statistically significant difference between different experimental groups (*p* < 0.05).

To evaluate T lymphocyte proliferation, we detected APPV E2-specific lymphocyte proliferative responses at 42 dpi. As shown in Figure 5, E2-IMS 1313VG and E2-ISA 201VG groups elicited proliferative responses. E2-IMS 1313VG and E2-ISA 201VG groups produced higher lymphocyte proliferative responses than those of the negative control group (*p* < 0.05). However, no significant difference in SI was observed between E2-IMS 1313VG group and E2-ISA 201VG group (*p* > 0.05). These results indicate that immunization of APPV E2 protein in mice can strengthen proliferative response and T cell activation.

### 3.5. Analysis of the Levels of Th1- and Th2-type Cytokines

**Figure 6:**
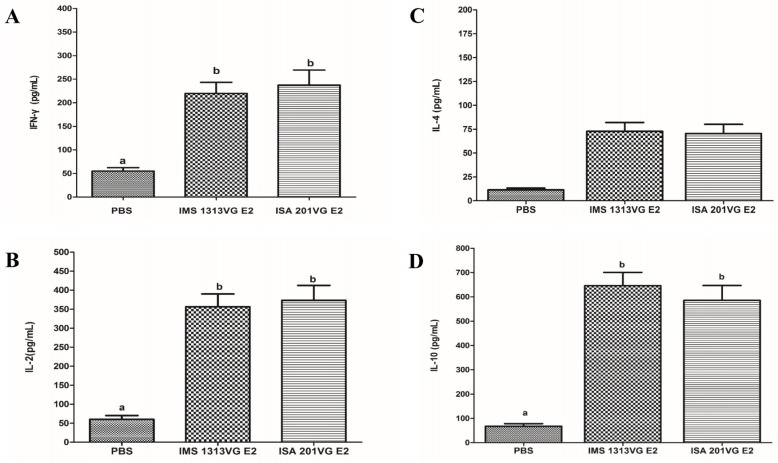
Detection of cytokine production in the supernatants of stimulated lymphocytes from mice immunized with the APPV E2 subunit vaccines. Concentrations of IFN-γ (**A**), IL-2 (**B**), IL-4 (**C**), and IL-10 (**D**) detected with commercial ELISA kits in accordance with the manufacturer’s instructions. Data represent the mean ± SEM. Different letters (a, b) indicate a statistically significant difference between different experimental groups (*p* < 0.05).

To characterize cellular immune response in the mice immunized with APPV E2 protein, we used a commercially available mouse IFN-γ, IL-2, IL-4, and IL-10 ELISA kit to determine the concentrations of Th1- and Th2-type cytokines in the medium supernatant of splenic lymphocytes stimulated with E2 protein. As shown in Figure 6A,B, the concentrations of IFN-γ and IL-2 (induced by Th1 cellular response) in E2-IMS 1313VG and E2-ISA 201VG groups were significantly higher than those of the negative control group. No significant difference was observed between E2-IMS 1313VG and E2-ISA 201VG groups (*p* > 0.05). The concentrations of IL-4 and -10 (induced by the Th2 cellular response) in Groups B and C were significantly higher than those of the negative control group (Figure 6C,D). Specifically, E2-IMS 1313VG and E2-ISA 201VG groups exhibited markedly higher IL-10 concentrations than those of other cytokines (*p* < 0.05). These results indicate that immunizing E2 protein can induce Th2-dominant cellular immune responses.

## 4. Discussion

Numerous CT cases have widely emerged in pig industries worldwide [2,3,6,7,8,9,10,11,12,13,14]. Recent research suggests that CT type A-II is associated with APPV, and CT type A-II is an important disease threatening the health of newborn piglets worldwide [1,2]. However, no effective vaccine nor drug can prevent or cure this disease. Vaccination is an effective method for preventing and controlling infectious diseases. Therefore, developing an effective vaccine for APPV is an urgent concern.

In our previous research, we demonstrated that the CSFV E2 subunit vaccine developed using baculovirus confers complete protection against CSFV [5]. On the basis of phylogenetic analysis, APPV has been considered a novel pestivirus [12,13,14]. E2 protein is the most abundant structural protein of APPV [3]. Bioinformatics analysis showed that APPV E2 protein features several B and T cell epitopes. Upon further analysis, we observed that the APPV E2 protein possesses three potential N-linked glycosylation sites (51N, 64N, and 103N; NetNGlyc 1.0 Server). In this study, the recombinant baculovirus Ac-3-APPV-E2 was successfully constructed. Western blot analysis revealed that the APPV E2 protein molecular weight reached 36 kDa, which is higher than the predicted molecular weight of APPV E2 protein (28.5 kDa). A possible explanation is that the APPV E2 protein is modified by glycosylation in insect cells. PNGase F was used to specifically remove N-linked glycans. Western blot analysis demonstrated that PNGase F can remarkably reduce the molecular weight of APPV E2 protein. Our study results indicate that the APPV E2 protein is a glycoprotein.

To obtain a secreted APPV E2 protein, we used an E2 gene containing the honeybee melittin signal peptide at the N terminus. Western blot analysis suggested that the expression product can be secreted into the culture supernatant. Then, the E2 protein was secreted in culture supernatant purified by the AKTA protein purification apparatus (GE, Stockholm, Sweden). The recombinant E2 protein was immunogenic in mice. Results of animal experiments showed that the APPV E2 protein emulsified with adjuvant can induce high levels of specific IgG antibody in mouse. The E2-ISA201VG subunit vaccine produced significantly higher antibody titers than the E2-IMS1313VG subunit vaccine. The high levels of the IgG1 antibody were induced by the E2-ISA201VG and E2-IMS1313VG subunit vaccines. The levels of IgG1 antibody were significantly higher than those of IgG2a and IgG3. The IgG1 antibody is a representative of Th2-type immune response. These results suggest that the APPV E2 subunit vaccine induces Th2 type-dominant immune response in mice.

Cellular immune response can play an important role in protecting against viral disease. The APPV E2-specific T lymphocyte proliferative response was detected in Groups B and C. The E2-ISA201VG subunit vaccine and E2-IMS1313VG subunit vaccine can yield a high SI value. The E2-ISA201VG and E2-IMS1313VG subunit vaccines can induce high levels of production of IFN-γ, IL-2, IL-4, and IL-10, especially production of IL-10, which is markedly higher than those of other cytokines. Results indicate that the Th2 type-dominant cellular immune response was induced in mice. This result is consistent with the subtype IgG results.

## 5. Conclusions

In conclusion, the recombinant baculovirus Ac-3-APPV-E2 was successfully constructed. Our findings demonstrate that the APPV E2 protein is a glycoprotein. The APPV E2 glycoprotein can induce a robust humoral immune response and cellular immune response in mice and Th2-type immune response. Our results show that the APPV E2 glycoprotein can be developed as a safe and effective subunit vaccine for the control of APPV infection. Further studies will be conducted to evaluate the immunogenicity of the APPV E2 subunit vaccine in pigs.
